# Can the outcome of post partum cardiomyopathy be predicted using late gadolinium enhancement CMR?

**DOI:** 10.1186/1532-429X-11-S1-P165

**Published:** 2009-01-28

**Authors:** Isabelle Roussin, Chirine Parsai, Sanjay Prasad, Dudley Pennell

**Affiliations:** Royal Brompton Hospital, London, UK

**Keywords:** Left Ventricular Ejection Fraction, Myocardial Inflammation, Late Gadolinium Enhancement, Myocardial Fibrosis, Enhancement Image

## Introduction

Post partum cardiomyopathy (PPCM) can occur between the last trimester of pregnancy or within 5 months post partum, in the absence of any history of heart disease. Although rare it may become unresponsive to medical treatment and can in such cases require mechanical support, or even heart transplant. Predicting its evolution is unfortunately not possible. Late gadolinium enhancement CMR can identify myocardial fibrosis and has shown its prognostic significance in DCM. To our knowledge the role of late gadolinium enhancement in PPCM as a predictor of outcome has not been reported.

## Purpose

To our knowledge the role of late gadolinium enhancement in PPCM as a predictor of outcome has not been reported.

## Methods

From 2003 to 2008, 9 women (mean age 38 +/- 5 years) with suspected PPCM, clinically or by echocardiography, underwent standard CMR with late gadolinium enhancement images (LGE) following Gd-DTPA injection at the time of PPCM suspicion and after a follow up period of 27 ± 15 months. 2 patients had no long term follow up (one having a new pregnancy, and one lost to follow-up).

## Results

The initial study showed no LGE in all 9 patients, and this was confirmed at long-term follow-up (7/9 patients) indicating absence of detectable myocardial fibrosis, infiltration or infarction. CMR functional changes found at the time of diagnosis and at follow up were respectively: Left ventricular ejection fraction (LVEF) 53 ± 8.1% versus 56 ± 5.7 % at follow-up. LVEDIV 108.3 ± 29.3 ml/m^2^ versus 99 ± 19.3 ml/m^2^, LVESIV 68 ± 44.2 ml/m^2^ versus 40 ± 13 ml/m^2^, LV mass index 82.3 ± 27 g/m^2^ versus 87 ± 37.7 g/m^2^, RVEDV 159 ± 43 ml versus 149 ± 22.4 ml, RVESV 75 ± 14 ml versus 64.1 ± 17.8 ml, RVEF 49 ± 11% versus 58 ± 9%. In addition, none of the patients showed active myocardial inflammation or oedema. At follow-up, 40% (n = 2/5) with previously impaired LV function normalised their LVEF. In 3/5 pts with biventricular dysfunction (mean LVEF: 48 ± 6%, mean RVEF: 38 ± 7%), all but one showed a complete recovery of RVEF and LVEF. In 1/7 pt, LVEF remained unchanged.

## Conclusion

In our cohort, lack of myocardial late gadolinium enhancement was not predictive of LV function recovery in PPCM suggesting that mechanisms other than myocardial fibrosis are involved in the genesis of ventricular dysfunction.

